# Metabolic profile in women differs between high versus low energy spenders during a low intensity exercise on a cycle-desk

**DOI:** 10.1038/s41598-022-14002-6

**Published:** 2022-06-15

**Authors:** Terry Guirado, Pierre Bourdier, Bruno Pereira, Elisa Le Roux, Audrey Bergouignan, Anthony Birat, Laurie Isacco, David Thivel, Martine Duclos, Lore Metz

**Affiliations:** 1 EA 3533, Laboratory of the Metabolic Adaptations to Exercise Under Physiological and Pathological Conditions (AME2P), BP 80026, UE3533, Clermont Auvergne University, 63170 Aubiere CEDEX, France; 2 Auvergne Research Center for Human Nutrition (CRNH), 63000 Clermont-Ferrand, France; 3 Department of Sport Medicine and Functional Explorations, Clermont-Ferrand University Hospital, G. Montpied Hospital, Clermont-Ferrand, France; 4 INRA, UMR 1019, Clermont-Ferrand, France; 5 Université de Strasbourg, CNRS, IPHC, UMR 7178, 67000 Strasbourg, France; 6 Division of Endocrinology, Metabolism and Diabetes, Anschutz Health and Wellness Center, University of Colorado, Anschutz Medical Campus, Aurora, CO USA; 7 Clermont-Ferrand University Hospital, Biostatistics Unit (DRCI), Clermont-Ferrand, France

**Keywords:** Occupational health, Public health, Risk factors

## Abstract

Active-desks are emerging strategies aiming at reducing sedentary time while working. A large inter-individual variability in energy expenditure (EE) profile has been identified and has to be explored to better optimize and individualize those strategies. Thus the present study aimed at comparing the metabolic and physical profile of individuals characterized as high spenders (H-Spenders) *versus* low spenders (L-Spenders) based on EE during a cycle-desk low intensity exercise. 28 healthy women working in administrative positions were enrolled. Anthropometric, body composition and fasting metabolic profile parameters were assessed. EE was determined by indirect calorimetry, at rest and during a 30-min cycle-desk use. Participants were categorized as H-Spenders and L-Spenders using the median of the difference between EE at rest and during the 30-min exercise. H-Spenders had higher mean EE (*p* < 0.001) and carbohydrate oxidation (*p* = 0.009) during exercise. H-Spenders displayed higher values for fasting plasma insulin (*p* = 0.002) and HOMA-IR (*p* = 0.002) and lower values for HDL-cholesterol (*p* = 0.014) than L-Spenders. The percentage of body fat mass was significantly higher in H-Spenders (*p* = 0.034). Individuals expending more energy during a low intensity cycling exercise presented a less healthy metabolic profile compared with L-Spenders. Future studies will have to explore whether the chronic use of cycle-desks during work time can improve energy profile regarding metabolic parameters.

## Introduction

Over the last century, the technological revolution (i.e. work automation, increase in transports use) led to tremendous changes in human behaviors favoring a global reduction in physical activities (PA) and an increase in sedentary behaviors (SB)^1^, particularly in high-income countries^2,3^. The independent and joint effects of those more recently adopted behaviors raise the risks of cardio-metabolic morbidity and all-cause mortality^2,4^. With the growth of desk-bound activities in the work environment, SB have taken an important part in individuals’ daily time^5^ resulting in a reduction in PA and total energy expenditure (EE)^6^. Active workstations (sit-to-stand, treadmill or cycle-desks) have been suggested as potential solutions to counterbalance the excessive amount of time spent seated at work^7,8^. Standing desks have been suggested to increase slightly but significantly EE at work (≈1.2 kcal.min^−1^)^9^ compared with sitting position. However, this strategy may not benefit everyone to the same extent; inter-individual variability has been previously reported in energy during a sit-to-stand protocol^10,11^. Some individuals displayed a significant increase in EE during a steady-state standing position compared to a sitting position, while only a small increase in EE was detected in others in response to this postural change. These previous results from Miles-Chan and al.^10,11^ raised questions regarding standing as an effective strategy to increase EE in the overall population. The variability in EE adaptation has been associated with some health parameters such as body fat mass that is positively correlated with the energy cost of standing posture in healthy inactive individuals^12^. Several studies have questioned the energetic cost of other different dynamic workstations such as walking on a treadmill or cycling desk^9,13^. While these studies obviously reported a substantial increase in EE (≈2–4 kcal.min^−1^) compared to seating position^9,14,15^, cycling desks have been suggested to be the best active workstation in terms of work and psychobiological performances^13^. Nevertheless, none of the studies investigating EE during cycle-desk utilization have identified the parameters that could explain these different energy profiles. Several authors have noticed that training status can influence cycling gross efficiency^16,17^, with higher trained subjects being more efficient (i.e. more thrifty). However, the exercise intensities used in these studies are moderate to high and might not be representative of EE adaptations during low intensity exercise on a cycle-desk. Hence, it remains unknown whether the energetic profile of individuals during low intensity activities such as cycle-desk can be explained by specific anthropometric, body composition, cardiometabolic parameters or physical fitness. Indeed, understanding the characteristics of individuals’ energetic profiles will enable a better optimization and individualization of active-desks strategies. In this context, the present study is the first to aim at comparing body composition, the cardiometabolic and physical fitness profile of individuals characterized as spenders *versus* non-spenders during a low intensity cycle-desk exercise, based on EE measurement. We hypothesized that participants with a more efficient energy profile will present healthier body composition, metabolic health and physical fitness.

## Methods

### Participants

Twenty-eight healthy women, administrative employees, with a body mass index (BMI) ranging from 18.5 to 29.9 kg/m^2^ and aged between 18 to 60 years, participated in the present study. To be included in the study participants had to: i) be engaged in less than 150 min of moderate-to-vigorous physical activity per week based on self-reported data; ii) declare having regular menstrual cycles; iii) not be pregnant or lactating; iv) be free of any cardiovascular or metabolic disorders; v) not be dieting; vi) be free of any medication (excepted oral contraceptive); and vii) have a stable body weight (< 3 kg change during the 6 months prior to screening). This study was approved by the French ethical committee (Comité de Protection Personne Ile De France VIII 19 09 66) and all methods were performed in accordance with the relevant guidelines and regulations. Written informed consent was obtained for all participants in the present study.

### Experimental design

**Figure 1:**
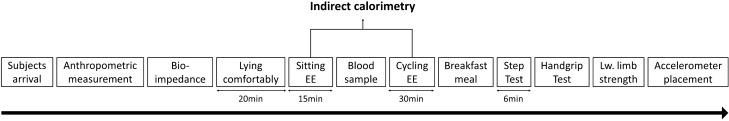
Schematic representation of the experimental design. EE, energy expenditure; Lw., lower.

After a full medical examination to assess eligibility, all subjects were asked to join the laboratory (laboratory AME2P, Aubière, France) for an experimental visit between January 6th and January 24th, 2020. Subjects were asked to keep their habitual daily activities, avoid any stressful situations and not consume caffeine for the 24 h prior to the test day. All participants completed this experimental session (Fig. 1) during the follicular phase of their menstrual cycle. Subjects reported to the laboratory at ~ 08.00 am, after a 12-h overnight fast. Evaluation started with body composition assessment and EE at rest was then investigated. Blood sample was obtained before a light intensity cycling exercise during which EE was measured. Participants’ physical fitness was evaluated on the same day after a standardized breakfast meal. Finally, before leaving the laboratory, participants received an accelerometer to be worn for the following 7 days in order to assess their daily physically active and sedentary time.

### Anthropometric measurement and body composition

Height was measured with a stadiometer at the nearest 0.1 cm, waist circumference (WC) was measured with a tape measure at the nearest 0.5 cm and WC to height ratio (WHtR) was calculated. Body weight was assessed using a calibrated scale (SECA, Les Mureaux, France) and fat mass percentage (%FM) and fat-free mass (FFM-kg) were evaluated by bioelectrical impedance (Tanita MC-780, USA, Arlington Heights), following the manufacturer’s instructions.

### Energy expenditure and substrate oxidation

After calibration of the device, indirect calorimetry with a facemask (MetaMax 3b, Cortex Biophysik, Leipzig, Germany) was used to measure VO_2_ and VCO_2_ for EE and substrate oxidation assessment. A heart rate monitor (Polar A300, Polar, Kempele, Finland) was used for the length of the experiment. Prior to resting condition subjects were sitting quietly for 15 min. For resting condition, subjects were lying comfortably in a deckchair in a thermoneutral environment for at least 20 min. After this period, subjects were asked to stay calm, not speak and avoid any movement. Gas exchanges were recorded for 15 min minimum and only the last 5 min were analyzed as previously suggested^18^ and were defined as “Rest” time measure.

During the exercise condition, subjects were submitted to a 30-min light exercise using a cycle-desk (DeskCycle, 3D Innovations LLC., Greeley, CO, USA) with a resistance set at 2 out of 8 per design of the ergometer and a revolution per minute (RPM) at 50 during the whole test, representing a power of ~ 16 Watts. An investigator supervised that participants respected the speed during the cycling test and reported at the end of the exercise the distance covered to ensure the test condition was similar between subjects. After the 30-min exercise testing, subjects had 1-min of recovery. Gas exchanges were measured during the entire exercise test and recovery period. EE, using Weir’s equation^19^, respiratory quotient (RQ; VCO_2_/VO_2_) and substrate utilization, using Péronnet & Massicotte equations^20^ were calculated for the whole 30-min exercise session and also at rest, and after 5 (Start), 10, 20, 30 min of exercise. Mean values of the last 2 min of each period were considered for analysis as done in previous studies^21^. The first minute of recovery was also considered for analysis.

### Cardiometabolic outcomes

Systolic and diastolic blood pressures were measured in a seated position using an auditory stethoscope with a blood pressure cuff adapted to the arm circumference. Subjects remained comfortably installed on a deckchair to collect a fasting blood sample. Plasma glucose, triglycerides, light-density lipoprotein cholesterol (LDL-cholesterol), high-density lipoprotein cholesterol (HDL-cholesterol) and total cholesterol were measured by enzymatic commercial assays. Insulin was assessed by chemiluminescent enzyme immunoassays. The enzymatic kits can be found in Supplemental file 1. All blood samples were centrifuged and plasma was kept frozen in aliquots at − 80 °C prior to analyses. The homeostasis model assessment of insulin resistance (HOMA-IR) was calculated by the following formula: fasting blood insulin (mU/L) x fasting blood glucose (mmol/L) / 22.5^22^.

### Physical fitness

#### Aerobic fitness

Participants performed a 6 min step test as described before^23^. Participants wore a heart rate monitor (Polar A300, Polar, Kempele, Finland) to continuously record heart rate from the start to the end of the test, 30 s and 1 min in recovery.

#### Upper and lower limb strength

Participants performed a handgrip test as described in previous studies^24^. Then, participants were seated with a hip joint at 105° of flexion and were attached on the trunk, the hip and the left leg to the dynamometer chair (Biodex System 2, Biodex, Shirley, USA) with Velcro straps. Torque was measured on isometric 3 s-Maximum Voluntary Contraction (MVC) and on concentric MVC at a velocity of 60°/sec and 120°/sec.

### Daily physical activity and sedentary time

From the day after the experiment, every subject was asked to wear triaxial accelerometers (ActiGraph wGT3X-BT, ActiGraph, Inc., Pensacola, FL) during 7 days with at least one weekend day. Participants wore the device on the right hip^25^ on an elastic belt. Data were collected at a frequency of 60 Hz and converted to counts per 1 s epoch using the manufacturer’s software (ActiLife version 6.13.4). Non wear time was defined as 90 min of 0 count per minute (cpm) with an allowance of 2 min of activity when it is placed between two 30-min windows of 0 cpm^26^. To be accepted in the analysis, accelerometer data had to be at least 4 days (including 1 weekend day) of wear with a monitor wear time of ≥ 10 h/day (600 min/day)^27^. SB was calculated with the vertical axis and PA with vector magnitude. SB was defined as < 150 counts min^−1^^28^, light intensity PA (LIPA) was obtained by subtracting SB and data below 2689 counts min^−1^, MVPA was defined as 2,690–6166 counts min^−1^, vigorous PA (VPA) was defined as < 6467 counts min^−1^^29^.

### Statistical analyzes

The sample size was estimated in order to compare the metabolic and physical profile of individuals characterized as high spenders (H-Spenders) versus low spenders (L-Spenders) based on EE during a cycle-desk low intensity exercise. To highlight significant differences greater than 1 point effect-size, 14 participants by group (H-Spenders vs. L-Spenders) were needed for 80% satisfactory statistical power and a two-sided type I error at 5%.

Statistical analysis was performed using Stata software (version 15, StataCorp, College Station, Texas, USA). Data were presented as mean and standard deviation. The Shapiro–Wilk test was used to test the assumption of distribution normality for quantitative parameters. Energy profile was determined by categorizing difference between EE at rest and 27 min of exercise (3-27 min) (Delta Exo-Rest) according to statistical distribution, i.e. to median of the sample^30,31^. This categorization enabled to have two different groups: High Spenders (H-Spenders) and Low Spenders (L-Spenders). The comparisons between groups (above versus below the median value), were performed by repeated-measures ANOVA and post-hoc Bonferroni test was used for multiple comparisons with significance levels set at p < 0.05. The statistical tests were two-sided, with type I error at 0.05. Then, a sensitivity analysis was conducted to guaranty that these analyzes realized according to median value were robust and that conclusions can be supported by the results. Delta Exo-Rest was categorized according to values ranged between interquartile ranges. The comparisons were performed as aforementioned. More precisely, for each value of Delta Exo-Rest between first and third quartile, continuous variables were compared among < or ≥ of each value of Delta Exo-Rest. The results were expressed as Hedges’ effect size (ES) and 95% confidence intervals, and were interpreted according to Cohen’s rules of thumb, which defines effect-size bounds as: small (ES: 0.2), medium (ES: 0.5) and large (ES: 0.8: grossly perceptible and therefore large). Multivariate analysis was conducted using multiple linear regression to adjust results on weight of participants. The assumption of residuals normality was analyzed as aforementioned. When appropriate, a logarithmic transformation was applied. As these analyzes could be considered as exploratory, individual p-values have been reported without applying any mathematical correction but with specific attention to the magnitude of differences (i.e. ES), according to several works reported in the literature like those discussed by Bender and Lange^32^. Furthermore, principal component analysis was also performed to investigate relationships between quantitative variables using R software (R Foundation for Statistical Computing, Vienna, Austria). This statistical method was useful for analyzing assets as elements of quantitative variables in order to i) uncover the underlying relationships and structures of the measured variables (latent constructs) and ii) to aggregate subjects into clusters such that each cluster represents a topic.

## Results

### Anthropometric, body composition and cardiometabolic outcomes

H-Spenders and L-Spenders were aged 37.7 ± 7.6 and 41.9 ± 10.9 y.o., respectively, with a mean BMI of 23.9 ± 3.8 and 21.6 ± 1.7 kg/m^2^. H-Spenders had a higher percentage of body fat mass (p = 0.034) and WHtR (p = 0.025) and lower fasting plasma concentration of HDL-C (p = 0.014) compared to L-Spenders (Table 1). A lower insulin sensitivity was observed for H-Spenders compared to L-Spenders, as indicated by greater plasma insulin concentrations (p = 0.002) and HOMA-IR (p = 0.002) values (Table 1). No other between-group significant difference was reported in body composition and cardiometabolic outcomes (Table 1).

**Table 1:** Characteristics of the study population.

Variables	Low spenders	High spenders
N	14	14
Age (years)	41.9 (10.9)	37.7 (7.6)
Height (cm)	164.4 (4.7)	163.8 (7.3)
Body weight (kg)	58.4 (4.6)	64.5 (11.8)
BMI (kg/m^2^)	21.6 (1.7)	23.9 (3.8)
Body fat mass (%)	25.9 (5.9)	31.5 (6.6)*
Body fat-free mass (kg)	40.9 (2.4)	41.4 (4.8)
Waist circumference (cm)	73.2 (6.0)	82.0 (11.3)
Waist circumference/height	0.44 (0.04)	0.50 (0.07)*
Systolic blood pressure (mmHg)	112.1 (5.8)	121.1 (14.9)
Diastolic blood pressure (mmHg)	70.0 (6.0)	76.1 (8.4)
Glucose (mmol/L)	4.79 (0.32)	5.12 (0.90)
Insulin (mIU/L)	4.04 (1.72)	9.25 (6.46)**
HOMA-IR	0.86 (0.36)	2.30 (2.35)**
Total cholesterol (g/L)	1.73 (0.45)	1.68 (0.25)
HDL-Cholesterol (g/L)	0.66 (0.09)	0.54 (0.13)*
LDL-Cholesterol (g/L)	0.99 (0.24)	1.00 (0.27)
Triglycerides (g/L)	0.83 (0.38)	0.72 (0.28)

BMI, body mass index; HOMA-IR, homeostasic model assessment of insulin resistance; HDL, high-density lipoprotein cholesterol; LDL, light-density lipoprotein cholesterol.

Values are presented as mean score (standard deviation) or percentage.

Boldface indicates statistical significance (*p < 0.05, **p < 0.01), respectively with Mann–Whitney test.

### Daily physical activity, sedentary time and physical fitness

As displayed in Table 2, no significant difference was observed between the two groups for aerobic fitness, upper and lower limb strength, total and segmented (by intensities) physical activity levels and sedentary time (Table 2). Based on the recorded physically active and sedentary time, our population can be considered sedentary and physically inactive^1^.

**Table 2:** Physical activity level, sedentary time and physical fitness of the study population.

Variables	Low spenders	High spenders
N	14	14
Valid days of accelerometer wear	5.7 (0.4)	6.0 (0)
Weekdays	3.9 (0.3)	4.0 (0)
Weekend days	1.8 (0.4)	2.0 (0)
Number of minutes of accelerometer data (min/day)	352.9 (64.3)	368.9 (55.4)
Sedentary time (%/daily waking hours)	87.8 (2.9)	87.9 (3.1)
Total physical activity (%/daily waking hours)	12.2 (2.9)	12.0 (3.1)
LPA (%)	3.1 (1.1)	3.4 (1.1)
MVPA (%)	7.9 (1.9)	7.7 (2.7)
VPA (%)	1.2 (0.5)	0.9 (0.4)
Handgrip dominant hand (kg)	29.1 (4.7)	29.3 (4.7)
Handgrip non-dominant hand (kg)	28.0 (4.8)	26.5 (5.0)
Rest heart rate step test (bpm)	65.8 (6.8)	74.8 (12.7)
Heart rate step test (bpm)	147.9 (18.0)	160.3 (16.7)
Heart rate step test + 30 (bpm)	124.5 (19.9)	136.0 (19.3)
Heart rate step test + 60 (bpm)	106.2 (20.1)	118.1 (17.6)
Isometric strength (nm)	136.1 (31.7)	131.1 (36.6)
Isokinetic power 60°/sec (w)	142.7 (33.2)	140.1 (34.0)
Isokinetic power 120°/sec (w)	236.5 (55.7)	215.1 (60.5)

LPA, light intensity physical activity; MVPA, moderate-to-vigorous physical activity; VPA, vigorous physical activity.

Values are presented as mean score (standard deviation) or percentage.

### Energy expenditure, heart rate and substrate oxidation

**Figure 2:**
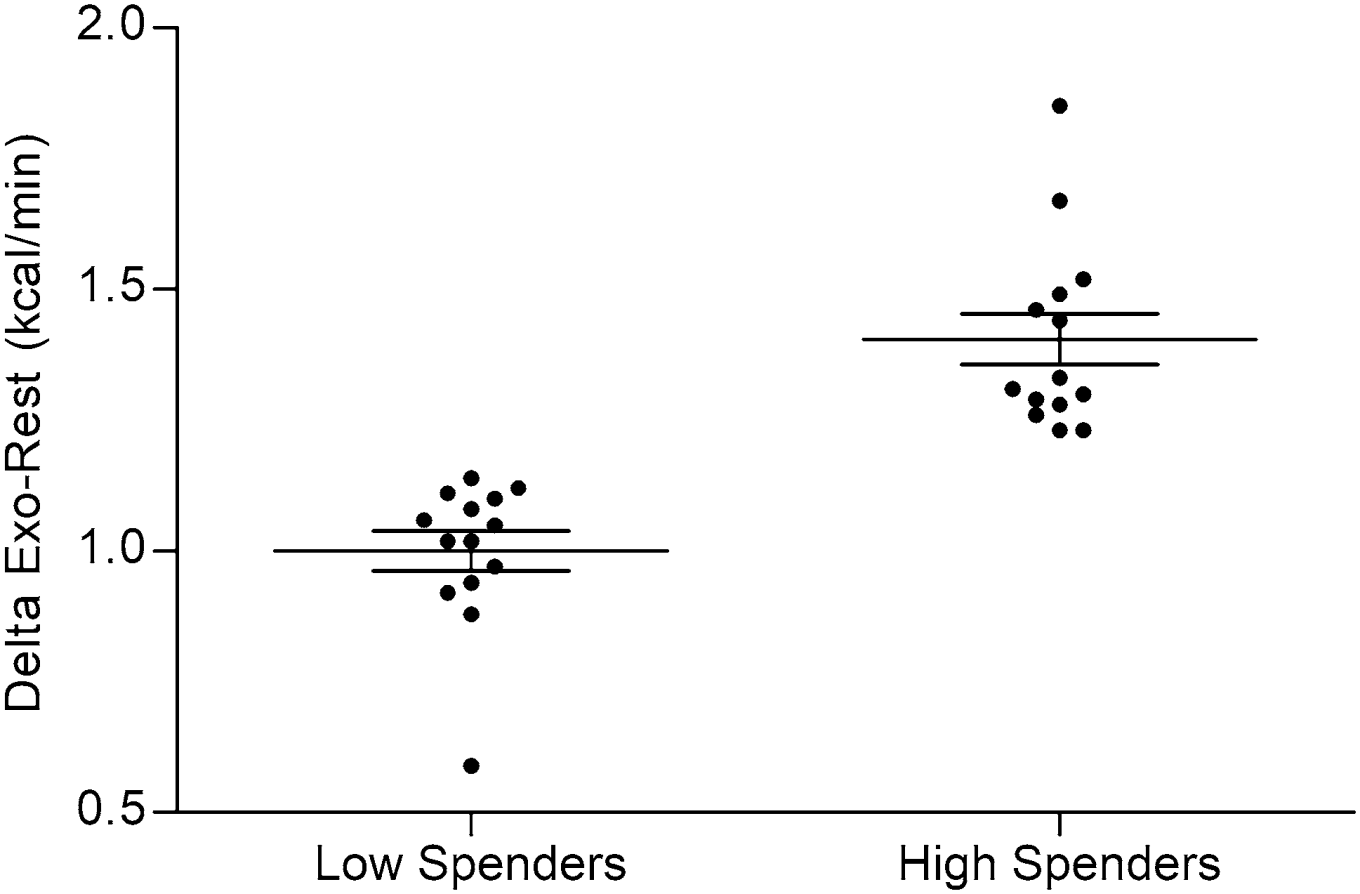
Characterization of Delta Exo-Rest between H-Spenders and L-Spenders. Data are presented as mean ± SEM.

**Figure 3:**
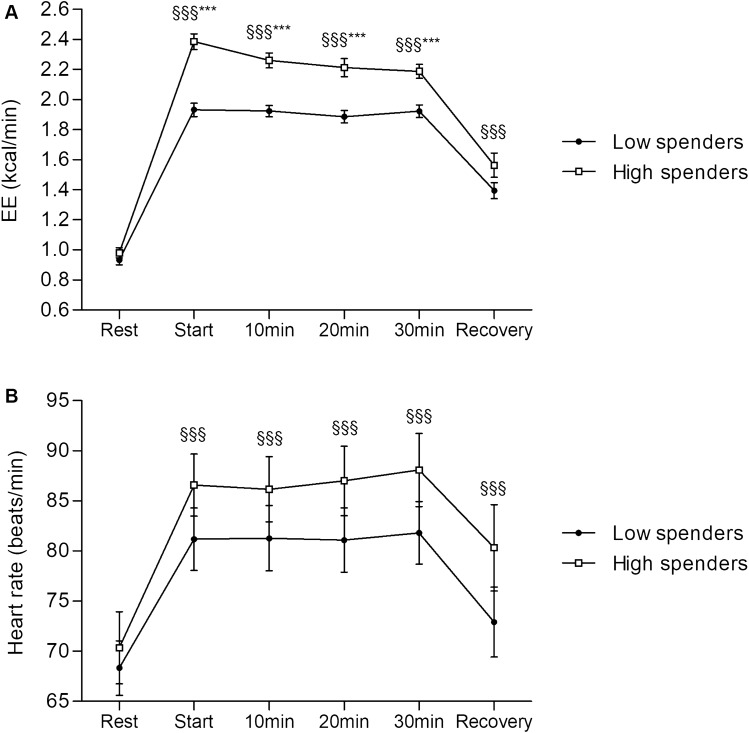
Comparison of EE (**A**), heart rate (**B**), from resting condition to light cycling exercise and recovery in each EE response group: H-Spenders and L-Spenders. Data are presented as mean ± SEM. §§§, time effect at p < 0.001; ***, significantly different between low spenders and high spenders at p < 0.001.

Overall, Delta Exo-Rest for EE showed large variability (0.5 to 1.8 kcal.min) (Fig. 2). Mean EE during the 30-min exercise increased significantly compared to mean resting EE in both H-Spenders (2.26 ± 0.2 *vs* 0.98 ± 0.12 kcal/min, p < 0.001) and L-Spenders (1.91 ± 0.15 *vs* 0.93 ± 0.11 kcal/min, p < 0.001). There was no between-group difference in EE at rest (0.98 ± 0.12 *vs* 0.93 ± 0.12 kcal/min, respectively). However, H-Spenders had higher EE than L-Spenders at every time point of the exercise test: start (2.38 ± 0.19 *vs* 1.93 ± 0.16 kcal/min, p < 0.001, respectively), 10 min (2.26 ± 0.18 *vs* 1.92 ± 0.13 kcal/min, p < 0.001, respectively), 20 min (2.21 ± 0.22 *vs* 1.88 ± 0.15 kcal/min, p < 0.001, respectively), and 30 min (2.18 ± 0.17 *vs* 1.92 ± 0.15 kcal/min, p < 0.001, respectively) (Fig. 3A). At 1 min-recovery, EE was not significantly different between the groups (1.56 ± 0.30 *vs* 1.39 ± 0.20 kcal/min, H-Spenders *vs* L-Spenders, respectively).

The light cycling exercise significantly increased heart rate compared to resting position in both H-Spenders (86 ± 11 *vs* 70 ± 12 beats/min, p < 0.001) and L-Spenders (81 ± 10 *vs* 68 ± 9 beats/min, p < 0.001) with no differences between the two groups. This increase was consistent across the entire duration of cycling for both groups (Fig. 3B).

**Figure 4:**
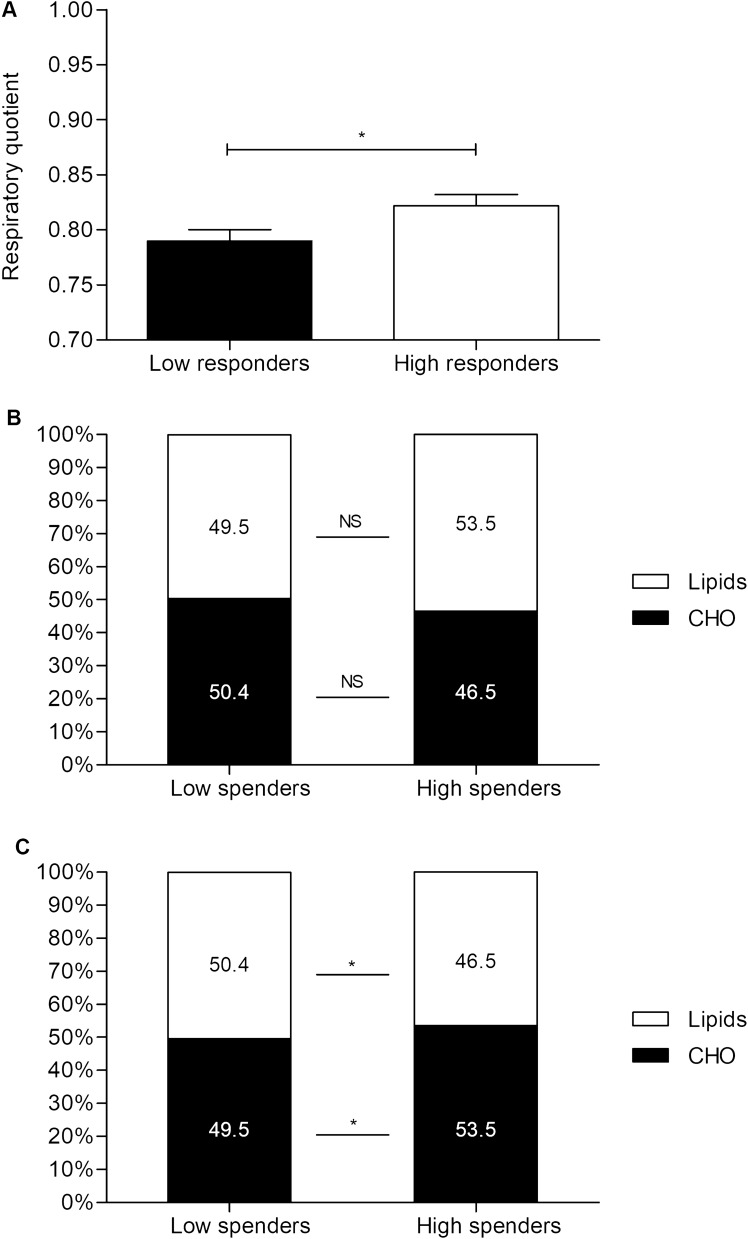
Respiratory quotient (**A**) during light cycling exercise. Substrate oxidation during Rest (**B**) and light cycling exercise (**C**). (**A**) data are presented as mean ± SEM. (**B**) and (**C**) data are expressed as mean percentage of CHO and lipids consumption relating to total energy expenditure. NS, not statistically significant; *, significantly different between low spenders and high spenders at *p* < 0.05.

RQ was similar between H-Spenders and L-Spenders at rest (0.84 ± 0.05 *vs* 0.83 ± 0.04, respectively) but was significantly higher in H-Spenders during the whole duration of exercise compared to L-Spenders (p = 0.021) (Fig. 4A). Taking all data together, there was a time effect (p = 0.009) for carbohydrates (CHO) oxidation, which was significantly higher during exercise compared to rest (p = 0.008) and recovery (p = 0.006). Resting CHO oxidation was significantly higher in H-Spenders compared to L-Spenders (3.33 ± 1.2 *vs* 2.82 ± 0.94 mg/min/kgFFM, p = 0.017, respectively). No significant difference was observed between groups at start, while H-Spenders oxidized significantly more CHO than L-Spenders during cycling at 10 min (6.52 ± 2.28 *vs* 4.53 ± 1.48 mg/min/FFM, p = 0.009, respectively), 20 min (6.52 ± 2.34 *vs* 4.79 ± 1.47 mg/min/kgFFM, p = 0.049, respectively) and 30 min (6.47 ± 2.15 *vs* 4.14 ± 1.38 mg/min/kgFFM, p = 0.008, respectively). No significant difference was reported at recovery between groups and compared to rest.

There was also a time effect for lipid oxidation, which was higher during exercise compared to rest for both H-Spenders (3.88 ± 1.55 *vs* 1.28 ± 0.33 mg/min/kgFFM, p < 0.001) and L-Spenders (3.85 ± 1.48 *vs* 1.27 ± 0.34 mg/min/kgFFM, p < 0.001). A group effect was noticed at the start of exercise and during recovery, with H-Spenders oxidizing more lipid than L-Spenders at start (5.94 ± 1.69 *vs* 4.76 ± 1.03 mg/min/kgFFM, p = 0.030, respectively) and oxidizing less lipid than L-Spenders during recovery (2.1 ± 0.44 *vs* 4.84 ± 2.34 mg/min/kgFFM, p = 0.002, respectively). No significant difference was reported for any other time of the exercise test.

Relative to total EE at rest, there was no significant difference in CHO oxidation in percentage between H-Spenders and L-Spenders (53.5 ± 17.8% *vs* 49.5 ± 14.9%, p = 0.26) or for lipid oxidation (46.5 ± 15.5% *vs* 50.4 ± 12.4%, p = 0.46) (Fig. 4B). During exercise, CHO oxidation was representing a greater percentage of total EE (44 ± 10.9% vs 35.7 ± 8.6%, p = 0.050) and lipid oxidation a lower percentage (56 ± 8.9% vs 64.3 ± 7.2%, p = 0.045) in H-Spenders compared to L-Spenders (Fig. 4C). No specific correlation were found between EE or substrate oxidation parameters and body composition, anthropometric data or blood parameters.

### Principal component analysis

**Figure 5:**
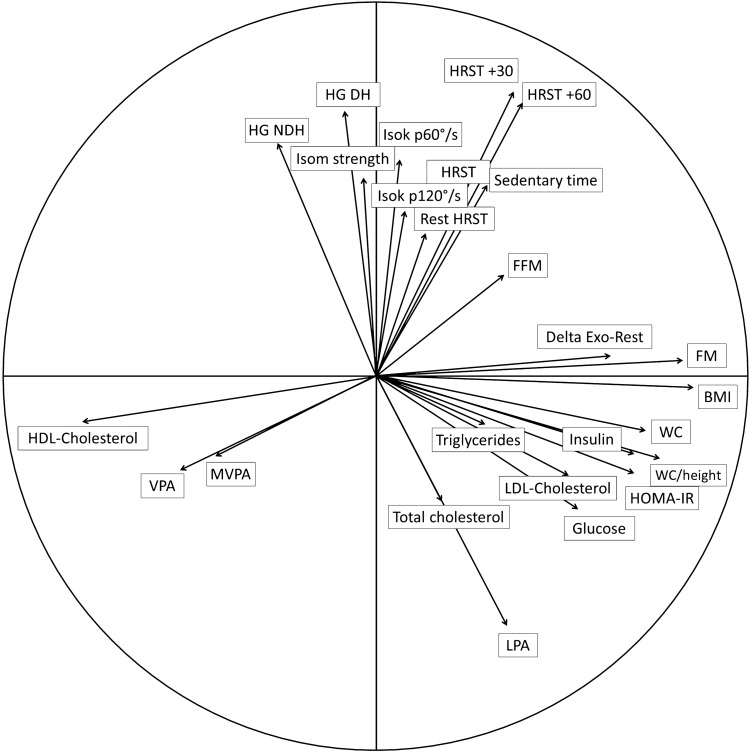
Principal component analysis of the study parameters. BMI, body mass index; DH, dominant hand; FFM, fat-free mass; FM, fat mass; HG, handgrip; HOMA-IR, homeostasic model assessment of insulin resistance; HDL, high-density lipoprotein cholesterol; HRST, heart rate step test; Isok, isokinetic; Isom, isometric; LDL, light-density lipoprotein cholesterol; LPA, light intensity physical activity; MVPA, moderate-to-vigorous physical activity; NDH, non-dominant hand; VPA, vigorous physical activity; WC, waist circumference.

Lastly, the associations between the different parameters studied were illustrated by a principal component analysis (Fig. 5). Our data has shown a strong correlation between Delta Exo-Rest and some cardiometabolic parameters, such as inulin, HOMA-IR, LDL-cholesterol, glucose and triglycerides (Fig. 5). Also, the variability of energy expenditure between rest and low intensity cycling was strongly associated with higher values of body composition and anthropometric parameters (fat mass, fat-free mass, BMI, WC and WC/height) (Fig. 5).

## Discussion

Active workstations are currently promoted to decrease office-related sedentary time and increase PA in a public health perspective. The aim of the present study was to examine associations between energy expenditure during a low intensity exercise on a cycle-desk device and body composition, cardiometabolic parameters and physical fitness of tertiary employees. Our data shows that two energetic profiles (H-Spenders or L-Spenders) can be identified in premenopausal women. More importantly, those two profiles show significant differences in anthropometric data, body composition (fat mass and WHtR) and metabolic outcomes (insulin, HOMA-IR, and HDL-Cholesterol), with H-Spenders presenting a less healthy metabolic profile.

Our results show that a light intensity cycle-desk exercise can significantly increase EE between 1.9 and 2.4 METs compared to resting. This result is in line with previous studies^14,33^ and demonstrates that light intensity cycling allows to increase EE above EE associated with sedentary activities (i.e. 1.5 METs). A number of studies have questioned the effect of cycle-desk use on EE^14,21,34^ but, none of them has looked for the potential factors that could explain this EE variability. Heterogeneity in energy responses has been reported in other studies from a sitting position to a steady-state standing position^10,11^ with individuals characterized as “energy-savers” or “energy-spenders”. While studies of Miles-Chan et al.^10^ reported only 18% of their subject having a significant increase in EE compared to sitting (increase > 5% resting EE), all subjects of our study significantly increased their EE during the low-intensity cycling session. Differences in the magnitude of responses between the two studies are likely explained by the higher energetic demand induced by cycle-desk used in the present study compared to the standing position alone (1.9 to 2.3 METs vs ~ 1.2 METs)^9^.

Light-intensity cycling was more demanding for H-Spenders who were eliciting higher EE at each period of exercise than L-Spenders. During exercise, H-Spenders oxidized more CHO, both in total amounts and relatively to EE, but a lower percentage of lipids compared to the L-Spenders, while H-Spenders had significantly more fat mass than L-Spenders. Relationships between fat mass percentage, body weight and substrate oxidation during exercise have been investigated in several studies with no clear association between these parameters^35,36^. Studies comparing substrate oxidation during exercise in women with normal weight and overweight did not show clear differences^35,36^. It suggests that excess of fat mass does not necessarily result in a decrease in the ability to oxidize lipids. However, fat mass localization in normal or overweighed subjects seems to be more associated with substrate oxidation during exercise^37,38^ than percentage of fat mass per se, with lower body fat mass profile being associated with better ability to oxidize lipids. In this line, we found that H-Spenders displayed higher %FM and WHtR, suggesting higher abdominal repartition of fat mass in individuals with this energy profile. The ability to rely predominantly on lipids or carbohydrates during submaximal exercise has been associated with the concept of metabolic flexibility, which is defined as the capacity to adjust fuel utilization to changes in fuel availability^39^. Metabolic state associated with glucose intolerance or insulin resistance has been shown to favor CHO oxidation during low intensity exercise compared to control subjects^40^ and has been associated with metabolic inflexibility^41^. The metabolic challenge induced in our study by a 30-min low intensity cycling exercise suggests that H-Spenders are less metabolically flexible than L-Spenders as they are less able to rely on lipids during a low intensity exercise^42^. Physical fitness and training status are also known to influence the ability to preferentially rely on lipids during low and moderate intensity exercise^43^. Thus, the H-Spenders and L-Spenders profiles could have been explained by differences in physical capacities of the subjects. This appears however unlikely here since heart rate during step test and higher and lower limb strength did not differ significantly between the two groups.

The potential mechanisms explaining heterogeneity in energy profile have been poorly investigated in previous studies questioning strategies to decrease SB during work time. Miles-Chan et al.^10^ did not find any association between body weight or height and EE when comparing energy cost in sitting *vs* standing positions. In a second study of the same research group, the energy cost of standing posture maintenance was positively correlated with body weight and WC^12^. Recently, Amaro-Gahete et al.^44^ showed that FFM could partly explain differences in EE profiles in sitting *vs*. standing position. Although H-Spenders had a higher percentage of fat mass, no difference in FFM was observed. These results are also concordant with the study of Chen et al.^45^, in which were reported relationships between energy efficiency and fat mass during walking, with subjects with obesity having decreased work efficiency compared to individuals with normal-weight during normal-speed walking. We further examined the cardiometabolic parameters of the two energy profiles. Our results suggest that H-Spenders showed a less healthy cardiometabolic profile as indicated by higher levels of fasting insulin, HOMA-IR and lower level of HDL-Cholesterol than L-Spenders. Metabolic profile and substrate oxidation during exercise of H-Spenders further feature similarities with those of subjects with obesity and/or type 2 diabetes^40^.

Individualization of exercise programs is a cornerstone of health management. Our results suggest that physical activity level and fitness capacities are not sufficient to discriminate people and that an energy evaluation at rest and during exercise should be assessed to personalize prescription. In light of our results, we can assume the H-Spenders benefit more from the same cycle-desk program than L-Spenders. Depending on the energy profile, it could be expected that cycle-desk use recommendations may need to differ in terms of time and/or intensity of pedaling. Given the increased demand and/or necessity in the utilization of active desks, this could have important implications for metabolic health management.

One potential limitation needs to be considered. We only studied women, thus those results are only applicable to the female population. However, Miles-Chan et al.^10^ reported different energetic profiles among male individuals during an activity at a lower intensity suggesting that the existence of different energy profiles might not be sex dependent. Nevertheless, the relation with body composition or metabolic profile could depend on this factor as shown by Chen et al.^45^. It is well known that hormonal status affects EE and two of our participants were taking oral contraceptives. Currently, there is no clear scientific evidence that oral contraceptives could induce modification of EE at rest or during exercise.

## Conclusion

This study confirms that light cycling exercise enables to increase EE compared to resting but, inter-individual heterogeneity exists in the magnitude of energetic response. Differences in physical fitness, habitual time spent active or sedentary are not explaining this inter-individual variability. However, female individuals who spend less energy during a low intensity cycling activity present a healthier metabolic profile than those who displayed higher EE. Identification of energy profile could represent a strategy to better individualize the use of dynamic workstations to optimize EE during workdays. Future studies will need to investigate whether long-term utilization of light-intensity cycling desk at work can improve metabolic health outcomes of sedentary office workers, especially those with less healthy metabolic profiles.

## Supplementary Information

Supplementary Information.
