# Supplementary material for: Metabolic profile in women differs between high versus low energy spenders during a low intensity exercise on a cycle-desk
Source: Sci Rep. 2022 Jun 15;12:9928. doi: 10.1038/s41598-022-14002-6 (PMC9200836; doi:10.1038/s41598-022-14002-6)
Supplement: Supplementary file 1 — Supplementary Information. [file 41598_2022_14002_MOESM1_ESM.docx]

| Supplemental File 1. Enzymatic kits used for cardiometabolic outcomes. | |
| --- | --- |
| Blood parameters | Enzymatic kits |
| Glucose | GLU Flex reagent cartridge* |
| Triglycerides | TRIG Flex reagent cartridge* |
| LDL-cholesterol | LDLC Flex reagent cartridge* |
| HDL-cholesterol | HDLC Flex reagent cartridge* |
| Total cholesterol | CHOL Flex reagent cartridge* |
| Insulin | Enzyme immunoassays** |
| LDL, light-density lipoprotein cholesterol; HDL, high-density lipoprotein cholesterol; *, Dimension Vista System, Siemens Healthcare Diagnostics Inc. 2008, Newark, USA; **, Siemens Immulite® 2000, Siemens Healthcare Diagnostics, Gwynedd, UK. | |
|  |  |
|  |  |
|  |  |
